# Vitamin D status in adults and children in Transcarpathia, Ukraine in 2019

**DOI:** 10.1186/s40795-020-00380-5

**Published:** 2020-11-06

**Authors:** Khrystyna Shchubelka

**Affiliations:** Department of Medicine, State University “Uzhhorod National University”, Pl.Narodna 1, Uzhhorod, 88000 Ukraine

**Keywords:** Vitamin D deficiency, 25-hydroxyvitamin D, Ukraine, Adults, Children

## Abstract

**Background:**

Vitamin D deficiency is a global health problem, it is assessed by measuring serum 25-hydroxivitamin D (25(OH) D), nevertheless epidemiological data for many countries remains underreported.

**Objectives:**

To study the prevalence of vitamin D deficiency throughout the calendar year in a large cohort recruited ina multiethnic Transcarpathian region of Ukraine.

**Methods:**

In this retrospective study 25(OH) D serum concentration was measured during all 12 months of the year 2019 by electrochemoluminescent assay on the automatic analyzer Cobas e411 in 1823 subjects, including both children and adults (1551 females (85.03%) and 273 males (14.97%)).

**Results:**

The mean 25(OH) D concentration in adults demonstrates significantly lower levels compared to children (22.67 ± 8.63 ng/ml vs. 26.00 ± 10.72 ng/ml respectively, *p < 0.001*). Adult women expressed significantly lower mean annual serum 25 (OH) D concentrations in comparison to men (22.29 ± 8.46 ng/ml vs. 25.75 ± 9.38 ng/ml respectively, *p < 0.001*). In contrast, children did not show a significant difference between sexes (girls 24.98 ± 10.38 ng/ml vs. boys 27.01 ± 11.01 ng/ml, *p = 0.2003*). In the winter months, 25(OH) D levels fell below 20 ng/ml in 51,74% of adult population of Thranscarpathia, and in 12.91%, − below 12 ng/ml.

**Conclusions:**

The results of this study contradict the previously reported evaluations of the vitamin D levels in Ukraine which were assessed by measuring serum 25(OH) D. Specifically, only approximately half of the studied population is vitamin D deficient during winter season. This study features the most representative sample size in Ukraine to date.

## Introduction

Vitamin D is an important fat-soluble vitamin that acts as a steroid prohormone playing a key role in bone mineralization. It is synthesized in skin under UV light exposure and is ingested in food [1]. To date, the effects of vitamin D have been associated not only with musculoskeletal disorders, but also with such frequently encountered pathologies as cancer, autoimmune, inflammatory, infectious and cardiovascular diseases, as well as diabetes [2]. Levels of vitamin D in the population depend on UV light exposure, consequently geographic location, skin type, clothing culture, testing methods, nutritional habits including supplements consumption, as well as genetic factors [3].

Vitamin D status is generally evaluated by measuring serum 25-hydroxyvitamin D (25(OH)D), the gold standard method for this test is being liquid chromatography-tandem mass spectrometry (LC-MS/MS) [4]. However, despite the vast knowledge amassed on the topic, the concentration of 25 (OH) D that indicates vitamin D deficiency in the human body still remains controversial. The National Academy of Medicine (USA) [5] considers levels of 25(OH) D below 12 ng/ml to be a sign of vitamin D deficiency, while The Endocrine Society (www.endocrine.org) [6] contends that the threshold level is at 20 ng/ml. Also, a concentration below 10 ng/ml is commonly defined as “severe deficiency” and indicates the risk of rickets and symptomatic osteomalacia development [7, 8].

Indisputably, vitamin D deficiency is a global health problem, which has been reported in many countries, particularly among institutionalized elderly, postmenopausal women, and immigrants. Reportedly, from 2 to 30% of Europeans may have levels of 25(OH) D below 10 ng/ml [3]. Interestingly, there is a sizable regional variation, as population of Nordic countries seem to have higher vitamin D levels, that may be attributed to the traditionally high consumption of fish liver oil [9].

Ukraine is the largest European territory that spreads over various climate zones, from temporal to subtropical. Previous studies have reported that as much as 37,3% of the Ukrainian population could bevitamin D deficient (< 10 ng/ml), which is a very high proportion, specifically when compared to other European countries [8, 10]. In this study we attempted to validate the previous studies, and to reevaluate the prevalence of vitamin D deficiency using a large and the most representative sample to date collected in a multiethnic Transcarpathian region (Transcarpathia) of Ukraine, bordering four other European countries (Poland, Slovakia, Hungary and Romania).

## Methods

This retrospective study included 1823 randomly selected subjects among those, whose serum concentration of 25(OH) D was recorded in 2019 at the medical laboratory center “Astra Dia” (www.astra-dia.ua) in Transcarpathian region, Ukraine. The recorded data was depersonalized and the following information was included: sex, age, and month of blood collection. The 25(OH) D serum concentration was measured on the automatic analyzer Cobas e411using the “Elecsys Vitamin D total” electrochemoluminescent assay (Roche®, Germany). To the best of our knowledge, as self-declared by individuals studied, no dietary supplements were consumed at the time of blood draw. The data was analyzed taking into account two established 25(OH) D concentration cut-offs indicating deficiency: < 12 ng/ml by National Academy of Medicine, USA and < 20 ng/ml by Endocrine Society, USA. All the statistical data analysis, including normality of the distribution (Shapiro-Wilk test), the differences between groups (2-sample independent T-test) and ANOVA (Analysis of variance) were performed using STATISTICA® software (StatSoftInc., Tulsa, OK, USA). *P* values less than 0.05 were regarded as statistically significant.

## Results

Among the 1823 studied individuals, 1551 were females (85,03%) and 273 were males (14,97%). The majority (1639 or 89,9%) were adults and only 184 (10,1%) were either children or adolescents (aged < 18 years). The average age (mean ± SD) among all the adults was40,13 ± 13,41 years (range 18–82), while the males were slightly older (42.21 ± 14.66, range 18–75 years) than the females (39.87 ± 13.22, range 18–82 years). Among the children and adolescents (< 18 years) the average age was10.67 ± 4.83 years, with males (9.3 ± 4.75 years) younger than females (12.05 ± 4.52 years). Both age and 25(OH) D concentration data were normally distributed (Shapiro–Wilk test, *p > 0.05*).

We analyzed crude 25(ОН) D levels every month of the calendar 2019 year among adults and children, and observed significant seasonal variability in 25-hydroxyvitamin D expression levels (ANOVA test, *p = < 0.001*). Among the adults the lowest levels were observed in February (19.44 ng/ml), and the highest - in September and July (26.97 ng/ml and 26.83 ng/ml, respectively). Similarly, children and the adolescentsexpressedthe lowest levelsof 25(ОН) D in February (16.60 ng/ml), and the highest in August (32.53 ng/ml). Monthly 25(OH) D concentrations in different age categories are illustrated in Table 1.

**Table 1:** Monthly levels of 25 (ОН) D in Transcarpathia, Ukraine

Month^a^	Adults		Children and adolescents	
	Mean ± SD 25(ОН)D (ng/ml)	Number of individuals	Mean ± SD 25(ОН)D (ng/ml)	Number of individuals
**January**	21.09 ± 8.74	125	22.56 ± 7.72	11
**February**	19.45 ± 7.67	174	16.60 ± 6.41	12
**March**	21.57 ± 9.18	130	25.43 ± 12.06	16
**April**	21.03 ± 8.44	127	23.56 ± 10.44	14
**May**	20.75 ± 7.59	151	19.80 ± 6.08	7
**June**	23.12 ± 8.18	137	26.02 ± 7.55	17
**July**	26.83 ± 9.56	139	29.59 ± 6.63	16
**August**	23.59 ± 7.71	125	32.53 ± 15.43	24
**September**	26.98 ± 8.00	129	27.66 ± 11.78	14
**October**	23.87 ± 8.59	134	27.48 ± 10.41	18
**November**	23.61 ± 8.83	136	25.86 ± 7.39	15
**December**	21.17 ± 7.58	132	24.76 ± 9.82	20
**Year mean**	22.67 ± 8.63	1639	26.00 ± 10.72	184

^a^ Measurements were conducted during 2019 and summarized for each month across the year

**Fig. 1:**
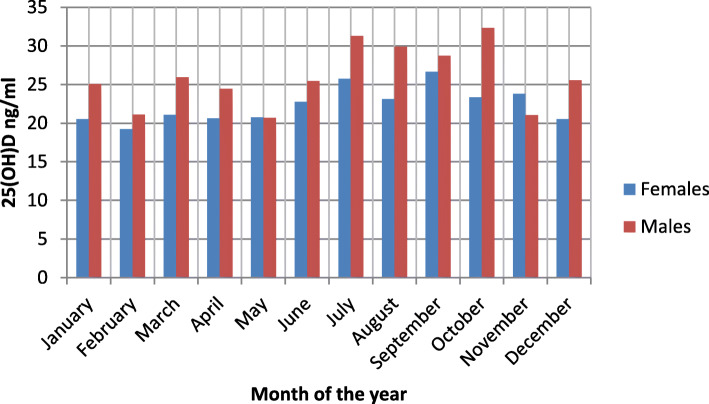
Monthly distribution of 25(OH) D concentration among adults presented by sex

In comparison to children and the adolescents, the adults had significantly lower mean annual serum 25(OH) D concentration (26.00 ± 10.72 ng/ml vs.22.67 ± 8.63 ng/ml respectively, *p < 0.001*). The adult women had significantly lower mean annual serum 25(OH) D concentration in comparison to the men (22.29 ± 8.46 ng/ml vs.25.75 ± 9.38 ng/ml respectively, *p < 0.001*). Monthly fluctuations of 25(OH) D concentration levels among adults presented by sex are indicated in Fig. 1. Statistical data was insignificant for sex dependence amongst children and adolescents (mean annual 25(OH) D in girls 24,98 ± 10.38 ng/ml vs. boys 27.01 ± 11.01 ng/ml, *p = 0.2003*).

The concentration of 25(OH) D negatively correlated with age both among adult males (r = − 0.16, *p < 0.05*) and females (r = − 0.12, *p < 0.05*). Strikingly, even stronger negative correlation of 25(OH) D with age was observed in children: among males (r = − 0.42, *p < 0.05*) and females (r = − 0.43, *p < 0.05*).

Since many studies calculate and report the prevalence of vitamin D deficiency in winter months [9] we also analyzed and utilized the winter data for comparison. In our sample, 51,74% of adult population of Thranscarpathia have 25(OH) D levels below 20 ng/ml and 12,91% - below 12 ng/ml in the winter (December through February of 2019). The prevalence of vitamin D deficiency in different sex and age groups in our sample is shown in Table 2.

**Table 2:** Prevalence of vitamin D deficiency during winter months (December–February 2019)

Indicator	Adult population	Adult males	Adult females	Children males	Children females
**Mean 25(ОН) D (ng/ml)**	20,45 ± 7,99	23,91 ± 8,33	20,09 ± 7,85	26,99 ± 8,98	20,98 ± 10.49
**Prevalence of 25(OH) D below 20 ng/ml**	51,74%	30,18%	54,64%	31,5%	58,3%
**Prevalence of 25(OH) D below 12 ng/ml**	12,91%	9,25%	13,52%	5,26%	20,83%

The prevalence of vitamin D deficiency in different age groups throughout the year is shown in Table 3. Across the age groups, the highest level of vitamin D deficiency is observed in adults over 60 years (58.7% of people with levels below 20 ng/ml, and 9.9% of people with levels below12 ng/ml). Among the children and adolescents, the highest prevalence of vitamin D deficiency is observed in the age range of 8–12 years old (41.86% below 20 ng/ml and 4.65% below 12 ng/ml). Vitamin D deficiency was completely absent in the infants and toddlers (0–3 years age group).

**Table 3:** Mean 25(OH) D concentration and the prevalence of vitamin D deficiency in different age groups in the calendar year

Indicator	Age categories								
	0–3 (n = 23)	4–7 (***n*** = 37)	8–12 (***n*** = 43)	13–17 (***n*** = 81)	18–25 (***n*** = 232)	26–35 (***n*** = 470)	36–45 (***n*** = 394)	46–60 (***n*** = 412)	> 60 (***n*** = 131)
**Mean ± SD 25(ОН) D ng/ml**	38.55 ± 11.25	29.55 ± 12.93	23.1 ± 8.01	23.33 ± 8.47	23.23 ± 8.13	24.18 ± 8.51	22.45 ± 9.21	21.76 ± 7.87	19.81 ± 9.35
**Prevalence of 25(OH) D below 20 ng/ml**	0%	21.62%	41.86%	35.8%	36.20%	35.10%	45.43%	44.1%	58.7%
**Prevalence of 25(OH) D below 12 ng/ml**	0%	2.7%	4.65%	0%	3.01%	3.40%	4.82%	5.8%	9.9%

## Discussion

To the best of our knowledge, this is the largest study of the vitamin D deficiency in Ukraine to date and the first study of this condition in the Transcarpathian region. Previously, the only countrywide study of vitamin D levels in Ukraine conducted and published in 2010 by Povoroznyuk et al. [10] reported that 81.8% of the Ukrainian population was deficient with levels of 25(OH) D below 20 ng/ml, while 37.3% had levels below 10 ng/ml. In addition, Povoroznyuk et al. [10] reported that the average level of 25(OH) D in Ukraine was 13.87 ng/ml, and the lowest level of 12.61 ng/ml was in western Ukraine, which geographically includes Transcarpathia as the westernmost region of the country. Unfortunately, the times of the year when the blood samples were drawn have not been reported for this study [10].

Overall, our data significantly differs from the results obtained by the other study discussed here; our results suggest a lesser prevalence of vitamin D deficiency than was thought previously. In contrast to the earlier study [10], our results indicate that only 51.74% of the studied population have a deficiency of vitamin D (below 20 ng /ml) in the winter months, and only 12.91% of individuals havelevels below 12 ng/ml in Transcarpathia. Moreover, in our sample the mean 25(OH) D concentrations during the winter months was at 20.84 ng/ml, while the annual average was at 22.67 ng/ml, almost twice the level previously reported [10].

There may be several reasons for the stark discrepancies in resultsbetween the two studies. First, Povoroznyuk et al. [10] reported using the electrochemoluminescent method, polyclonal Vitamin D assay (RocheDiagnostics®, Germany) on Elecsys 2010 analyzer. There were reports in 2011 that Roche Diagnostics® withdrew several lots of this particular assay from use referring to a deterioration of conformity to the reference method for the results reported (liquid chromatography - tandem mass spectrometry; LCMSMS), the same studies reported those polyclonal assays tend to lower the actual serum 25(OH) D levels [11]. In contrast, in the current study the samples were tested using the Elecsys assay with a newer generation kit containing a different analyzer (Cobas® e411, RocheDiagnostics®, Germany). Second, the season of sample collection can significantly contribute to the difference in our results. The exact difference between seasons cannot be established, due to the fact that the earlier study did not specify the timing of samples collection [10]. It is unlikely that the climate could account for a large difference, as Transcarpathia has similar average solar activity compared to other regions of Ukraine. The total annual amount of sun exposure in Transcarpathia lowlands is around 2000 h, while in mountainous areas it is approximately 1700 h, which is very similar to the total sun exposure in Ukraine overall (1700 to 2400 h annually) [12].

Furthermore, there could be a difference in the levels of 25 (OH) D in different studies due to different amount of sun exposure and dietary supplements intake by study participants. Some studies suggest that clothing practices (wearing a veil) or tendency to avoid sun may highly contribute to the vitamin D levels [13]. We did not access sun exposure by any means, but the patients have declared to have not taken any dietary supplements.

Given the close geographic proximity of Transcarpathia to other European countries it seems reasonable to compare our findings to those from neighboring countries. The similar values reported for these countries give additional credence to our results. For instance, a recent study in Poland that involved 5775 adults with a mean age of 54.0 ± 15.9 years reportedthe mean level of 25(OH) D at 18.0 ± 9.6 ng/ml [14]. This study also indicates that 65.8% of the population had a level of 25(OH) D below 20 ng/ml, which is closer to our results (51.74%) than to the earlier study of the Ukrainian population overall (81.8%). In addition, while our results showed lower levels in females in all age groups of adults in almost all months of the year, the study conducted in Poland showed lower levels of vitamin D in men compared to women. Although the authors believe the geographic location plays a minor role in vitamin D status in Poland, this may in fact account for the relatively higher prevalence of the deficiency in Poland (14.06% higher compared to Transcarpathia) because the territory of Poland is located more to the north (52.13° N,21.02° E) compared to Transcarpathia (48.41°N, 23.29°E). It should be noted that yet a different method of measurement of 25(OH) D was used in this Polish study (Liaison XL system (DiaSorin; CLIA method). The collection of material in this study was conducted from February 14 to March, and from April 28 to May 2, which is considered to be a “low” solar activity season. In the neighboring country Slovakia, a study of healthy women aged 25–40 years showed an average level of 25(OH) D at 32.6 ng/ml [15],which is 10.3 ng/ml above 22.3 ng/ml 25(OH) D reported for women in Transcarpathia during the year (mean age 39.87 ± 13.22 years). Unfortunately, this study did not account for the season. Similarly, the prevalence of severe vitamin D deficiency in Transcarpathia (12.91% below 12 ng/ml in the winter months) is lower than in Germany [16] and Great Britain [17], but higher than that reported in Spain [18], Italy [19], and France, where a study of 2007 individuals, in the age ranges 30–54 reported only 5,2%prevalence of vitamin D deficiency below 10 ng/ml [20]. The average level of vitamin 25(OH) D in adult population of Transcarpathia (20.84 ng/ml in the winter months) is closest to the levels reported in Austria (20.88 ng/ml, adults, age range 21–76 years) and Belgium (19.28 ng/ml, adults, age range 21–69, [21, 22]).

**Fig. 2:**
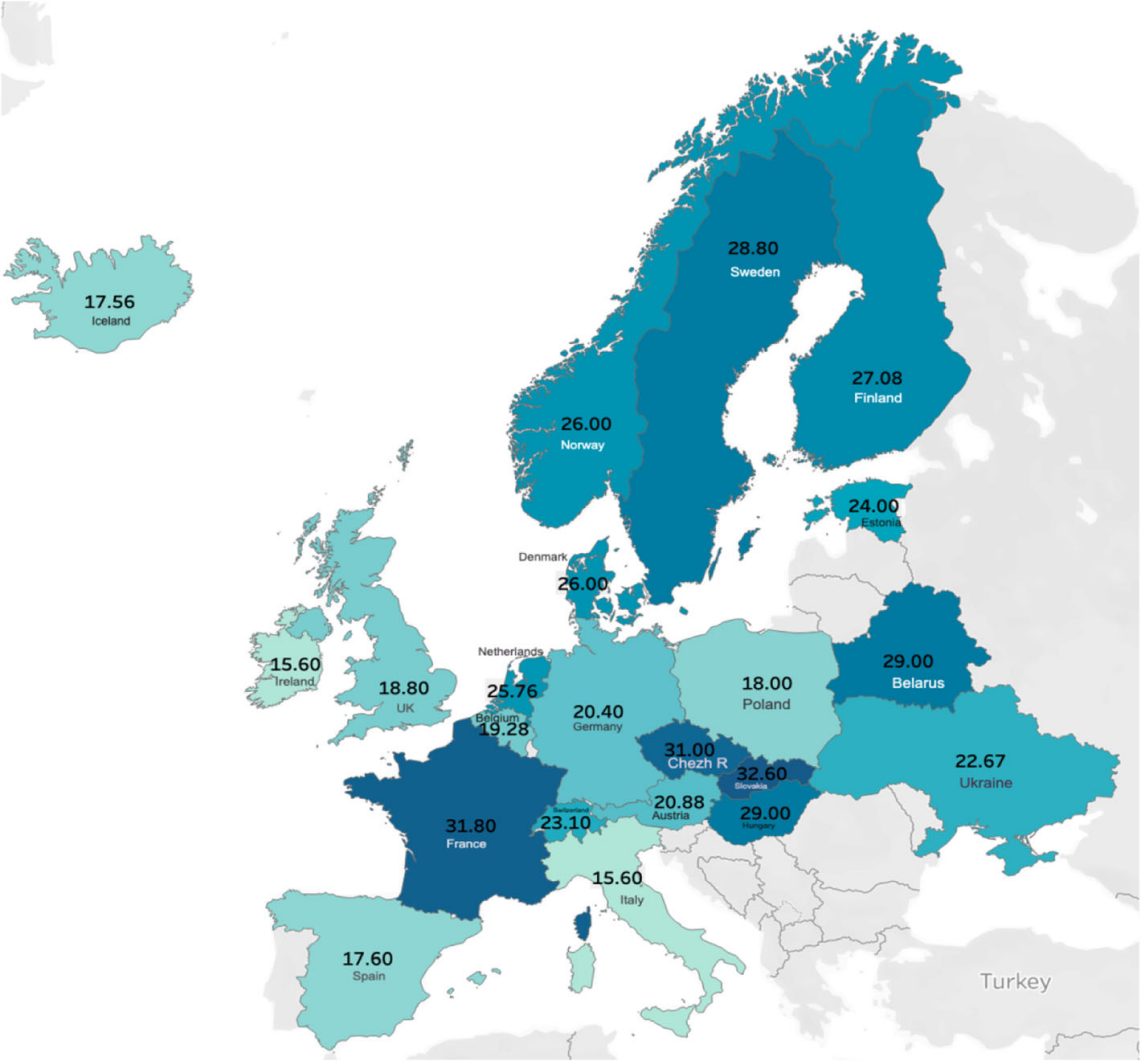
Mean 25(OH) D reported among adults in different European countries. This map is drawn by the author,using the software Tableau, data is taken from the cited studies, data for Ukraine is taken from our study

The above comparison supports the notion that Ukrainian population has similar levels of vitamin D deficiency when compared to its geographical neighbors, and that Transcarpathia may be part of the presumable north-south trend in serum vitamin D, where southern European countries have lower levels than northern European countries, though this trend does not follow the exact pattern (Fig. 2, [9, 14, 16–27]).

### Limitations

While our study had several limitations that prevented the direct comparisons to others (the method of measurement we used in our study is not the gold standard method for this type of test though it shows the correlation with tandem mass-spectrometry of r = 0,89 according to the manufacturer), and can be considered a rough population-based study on vitamin D status in the region, it highlights an important geographical trend that should be further explored and could be useful to aid the development of health care strategies in the region. Unfortunately, while we collected the seasonality data, we did not obtain data on the exact place of residence, and the origin of the people who were included in the study. Furthermore, the information on supplements consumption was self-reported. Also, our sample cannot be considered as a completely accurate representation of the general population, as we sampled a highly disproportional number of men and women, unfortunately this is due to the lack of testing of 25 (OH) D amongst males in general, in the region and in the country as a whole, and to a lesser extent, a lower number of males comparatively to females living permanently and temporarily in the country due to socio-economic reasons. All these could be useful covariates in the future investigations.

## Conclusions

This study showed a noticeable prevalence of deficiency of vitamin D in the Transcarpathian population in Ukraine, documented by a moderate decreasein 25(OH) D levels (< 20 ng/ml) in approximately half of the tested individuals in winter months. Only a limited part of the studied population (12.91%) expressed severely decreased 25(OH) D levels (< 12 ng/ml). Taking together, our findings contradict the previous study conducted in Ukraine, which showed much higher prevalence of vitamin D deficiency, but are consistent with the majority of the regional European reports on vitamin D status, thus fitting in the hypothetical south to north trend in deficiency across Europe. Adult women have significantly lower vitamin D levels than men, and children have higher levels than adults in our cohort (*p < 0,001).* Serum 25(OH) D concentration negatively correlates with age both in children and adults.

## Data Availability

The datasets used and/or analyzed during the current study are available from the corresponding author on reasonable request.

## Abbreviations

25(OH)D: 25-hydroxyvitamin D
ANOVA: Analysis of variance

## References

[1] Bhattoa HP (2017). Vitamin D: musculoskeletal health. Rev Endocr Metab Disord.

[2] Rosen CJ (2012). The nonskeletal effects of vitamin D: an Endocrine Society scientific statement. Endocr Rev.

[3] Lips P (2007). Vitamin D status and nutrition in Europe and Asia. J Steroid Biochem Mol Biol.

[4] Zerwekh JE (2008). Blood biomarkers of vitamin D status. Am J Clin Nutr.

[5] Ross AC (2011). The 2011 report on dietary reference intakes for calcium and vitamin D from the Institute of Medicine: what clinicians need to know. J Clin Endocrinol Metab.

[6] Holick MF (2011). Evaluation, treatment, and prevention of vitamin D deficiency: an Endocrine Society clinical practice guideline. J Clin Endocrinol Metab.

[7] Kennel KA, Drake MT, Hurley DL. Vitamin D deficiency in adults: when to test and how to treat. Mayo Clin Proc. 2010;85(8):752-8. 10.4065/mcp.2010.0138. 10.4065/mcp.2010.0138 PMC2912737 20675513

[8] Spiro A, Buttriss J (2014). Vitamin D: an overview of vitamin D status and intake in E urope. Nutr Bull.

[9] van Schoor, N. and P. Lips, Worldwide vitamin D status, in Vitamin D. 2018, Elsevier. p. 15–40.

[10] Поворознюк В, et al. Дефіцит та недостатність вітаміну D у жителів України. Боль. Суставы. Позвоночник. 2011;(4):5–12.

[11] Connell A (2011). Overreporting of vitamin D deficiency with the Roche Elecsys vitamin D3 (25-OH) method. Pathology.

[12] Масляк, П. and П. Шищенко, Географія України: пробний підруч. для 8–9 кл. серед. шк. К.: Зодіак-Еко, 2000.

[13] Batieha A (2011). Vitamin D status in Jordan: dress style and gender discrepancies. Ann Nutr Metab.

[14] Płudowski P (2016). Vitamin D status in Poland. Pol Arch Med Wewn.

[15] Vanuga P, et al. Vitamin D levels in young healtly premenopausal females in Slovakia. In 11th European congress of endocrinology. BioScientifica. Endocrine Abstracts. 2009;20:266. https://www.endocrine-abstracts.org/ea/0020/ea0020p266.

[16] Hintzpeter B (2008). Vitamin D status and health correlates among German adults. Eur J Clin Nutr.

[17] Adams J, White M (2015). Characterisation of UK diets according to degree of food processing and associations with socio-demographics and obesity: cross-sectional analysis of UK National Diet and nutrition survey (2008–12). Int J Behav Nutr Phys Act.

[18] Adami S (2009). Vitamin D status and response to treatment in post-menopausal osteoporosis. Osteoporos Int.

[19] Almirall J (2010). Association of low serum 25-hydroxyvitamin D levels and high arterial blood pressure in the elderly. Nephrol Dialy Transplant.

[20] Castetbon K (2009). Dietary intake, physical activity and nutritional status in adults: the French nutrition and health survey (ENNS, 2006–2007). Br J Nutr.

[21] MacFarlane G (2004). Hypovitaminosis D in a normal, apparently healthy urban European population. J Steroid Biochem Mol Biol.

[22] Kudlacek S (2003). Assessment of vitamin D and calcium status in healthy adult Austrians. Eur J Clin Investig.

[23] Kristinsson J, Valdimarsson Ö (1998). Serum 25-hydroxyvitamin D levels and bone mineral density in 16–20 years-old girls: lack of association. J Intern Med.

[24] McCarthy D (2006). Vitamin D intake and status in Irish elderly women and adolescent girls. Ir J Med Sci.

[25] Malvy DJM (2000). Relationship between vitamin D status and skin phototype in general adult population. Photochem Photobiol.

[26] Adami S (2008). Relationship between serum parathyroid hormone, vitamin D sufficiency, age, and calcium intake. Bone.

[27] Pludowski P, et al. Vitamin D status in central Europe. Int J Endocrinol. 2014;2014:13. https://www.hindawi.com/journals/ije/2014/589587/. 10.1155/2014/589587 PMC3984788 24790600

